# Antioxidant Defense and Heavy Metal Tolerance in *Adesmia subterranea*: Physiological Insights into Cadmium and Mercury Phytostabilization

**DOI:** 10.3390/plants15213392

**Published:** 2026-11-02

**Authors:** Victoria Parera, María Verónica Pérez Chaca, Camila V. Gatica Aguilar, Carlos A. Parera, Gabriela E. Feresin

**Affiliations:** 1 Instituto de Biotecnología, Facultad de Ingeniería, Universidad Nacional de San Juan, Av. Libertador General San Martin 1109 Oeste, San Juan 5400, Argentina; gferesin@unsj.edu.ar; 2 Consejo Nacional de Investigaciones Científicas y Técnicas (CONICET), CCT CONICET San Juan, Av. Libertador General San Martín 1109 (O), San Juan CP 5400, Argentina; caamigatica@gmail.com; 3 Facultad de Química, Bioquímica y Farmacia, Universidad Nacional de San Luis, Ejército de los Andes 950, San Luis 5700, Argentina; veroperezchaca@gmail.com; 4 Departamento de Biología, Facultad de Ciencias Exactas, Físicas y Naturales, Universidad Nacional de San Juan, Av. Ignacio de la Roza 590 (Oeste), Complejo Universitario Islas Malvinas, Rivadavia, San Juan 5402, Argentina; parera1956@gmail.com

**Keywords:** native species, heavy metal stress, Fabaceae, oxidative stress, phytostabilization

## Abstract

Heavy metal (HM) pollution is a major threat to terrestrial ecosystems. Addressing it requires identifying native plant species that tolerate HMs while maintaining physiological function. This study evaluated the physiological responses, antioxidant defense mechanisms, and phytoremediation potential of the high-Andean species *Adesmia subterranea* Clos under cadmium (Cd 3, 4.5, and 6 mg L^−1^) and mercury (Hg 0.8, 1.2, and 1.6 mg L^−1^) in a semi-hydroponic system. Survival, growth performance, metal accumulation, bioconcentration factor (BCF), translocation factor (TF), oxidative stress biomarkers such as hydrogen peroxide (H_2_O_2_), and antioxidant responses including catalase (CAT), ascorbate peroxidase (APX), and glutathione (GSH) were assessed. Exposure to Cd reduced survival and decreased biomass in a concentration-dependent manner, whereas Hg did not affect survival and reduced biomass only at 0.8 mg L^−1^. Both metals were efficiently taken up (BCF > 1); Cd was consistently retained in roots (TF < 1), while Hg translocation increased with dose (TF up to 0.79 at 1.6 mg L^−1^). Cd and Hg exposure induced oxidative stress, evidenced by increased H_2_O_2_ accumulation and lipid peroxidation, which was accompanied by activation of antioxidant defenses. The APX activity and GSH levels increased significantly, while CAT showed a metal-specific response. These results indicate coordinated regulation of enzymatic and non-enzymatic antioxidant pathways to maintain redox homeostasis under HM stress. Overall, these results provide a preliminary indication of the potential of *A. subterranea* for phytostabilization in metal-contaminated Andean ecosystems.

## 1. Introduction

Heavy metal (HM) pollution is one of the most challenging issues and a major driver of environmental degradation [1,2]. In the Central Andean region of Argentina, gold and copper mining operations are located at elevations between 2500 and 5000 m above sea level [3]; the large quantities of tailings and waste rock generated at these sites can lead to the accumulation of cadmium (Cd), copper (Cu), nickel (Ni), lead (Pb), mercury (Hg), zinc (Zn), and arsenic (As) in the surrounding soils [4]. Among these contaminants, Cd and Hg are of particular concern given their high toxicity even at low concentrations, their mobility in soil–plant systems, and their capacity to bioaccumulate across trophic levels [5,6].

Conventional approaches to HM-contaminated sites, including soil excavation, washing, and chemical immobilization, have been widely applied to address HM contamination in mining-impacted environments. However, their applications are limited due to high cost and energy requirements, safety hazards, and the risk of secondary ecological damage [7,8]. Against this background, biological alternatives such as phytoremediation have become a sustainable, low-cost technology that exploits the capacity of plants to take up, immobilize, or degrade contaminants [9].

Two main strategies are recognized for the phytoremediation of mine tailings and associated waste materials. Phytoextraction relies on the uptake and translocation of HMs into harvestable above-ground biomass, which is subsequently removed from the site for metal recovery or safe disposal [10]. Phytostabilization, in contrast, aims to reduce HM mobility and bioavailability through root accumulation or rhizosphere immobilization, thereby limiting contaminant dispersal through wind erosion or water runoff [11].

Cadmium and mercury exposure generates an imbalance between reactive oxygen species (ROS) production and the antioxidant capacity of biological systems, which can lead to macromolecular damage [12,13]. To counteract these effects, plants have developed an enzymatic and non-enzymatic antioxidant defense system. In this context, enzymes such as catalase (CAT) and ascorbate peroxidase (APX), as well as non-enzymatic molecules such as glutathione (GSH), actively participate in ROS detoxification, maintaining cellular redox homeostasis [14]. The integrated evaluation of oxidative stress indicators, including malondialdehyde (MDA) and hydrogen peroxide (H_2_O_2_), alongside antioxidant defense parameters constitutes a key tool to understand plant physiological responses to HM stress and assess species tolerance capacity [15].

Plant species selection is crucial for the success of phytoremediation programs. The species must be capable of tolerating heavy metal stress while maintaining adequate growth performance under contaminated conditions. The use of native species offers significant advantages, including improved local adaptation, greater seed and seedling availability, and reduced risk of biological invasion [16,17]. In this regard, species of the genus *Adesmia* (Fabaceae), restricted to arid and semi-arid high-Andean environments above 3000 m a.s.l. in Chile and Argentina, have been increasingly recognized for their ability to accumulate and stabilize HMs [18,19,20,21], highlighting the relevance of this genus for restoring metal-contaminated high-altitude environments. Moreover, because high-Andean species are naturally exposed to multiple environmental stresses such as drought and low temperatures [22], it is plausible that they possess stress-integration mechanisms that also support tolerance to metal stress [23].

Despite the growing mining activity in the high-Andean regions of Argentina, information on native species with phytoremediation potential for these environments remains scarce. In a previous study, we characterized the tolerance of *Adesmia subterranea* Clos and *Adesmia pinifolia* Gillies ex Hook. and Arn. to Cd, Hg, Ni and As during germination and early seedling growth, identifying Hg as the least phytotoxic metal, even acting as a stimulatory factor on root growth and biomass at this early stage [3].

Therefore, the present study extends this assessment to a later development stage, evaluating the physiological and biochemical responses of *A. subterranea* plants after prolonged exposure to Cd and Hg stress, with particular emphasis on antioxidant defense mechanisms and oxidative stress biomarkers. In addition, the capacity of this species to accumulate and translocate both metals was determined, in order to assess its suitability as a potential phytoremediation candidate for heavy metal-contaminated soils in semi-arid Andean environments.

## 2. Results

### 2.1. Survival, Plant Growth and HM Accumulation

Eight weeks of Cd exposure significantly decreased survival in *A. subterranea* (*p* = 0.0005), showing reductions of 13–21.5% compared with the control (Table 1), although survival did not differ among Cd concentrations. Consistently, both shoot and root biomass decreased in a concentration-dependent manner (*p* < 0.0001), as detailed in Table 1.

However, a different behavior was observed under Hg treatments. Survival remained stable across concentrations (81–83%), with no significant differences from the control (*p* = 0.1112; Table 2). Biomass responses varied by organ type (two-way ANOVA, *p* = 0.0462). At 1.2 mg L^−1^ Hg, shoot biomass reached its highest value (18.54 mg), comparable to the control, whereas at 0.8 mg L^−1^ Hg it showed the lowest value, representing an 11.9% reduction. Root biomass was generally comparable to the control, except for a significant reduction at 0.8 mg L^−1^ (Table 2).

Cd accumulation was determined in shoots and roots of *A. subterranea* to evaluate the metal distribution pattern between organs. The species exhibited differential Cd accumulation between organs, with root concentrations higher than those recorded in shoots across all treatments. Shoot Cd content increased significantly with rising metal supply (*p* = 0.0011), though the response showed a marked threshold effect, with no significant difference between the two lower treatments. A similar dose-dependent pattern was observed in roots, where Cd concentrations ranged from 1016.3 ± 115.1 µg g^−1^ to 2885.4 ± 306.0 µg g^−1^ (Table 3).

Table 3 summarizes bioconcentration factor (BCF) and translocation factor (TF) across the different Cd treatments. BCF values in both roots and shoots were consistently >1 across all Cd treatments, confirming effective accumulation. Notably, BCF values were more markedly elevated in roots (338.7–480.9) relative to shoots (53.8–76.0) (Table 3). In contrast, TF values remained <1 and did not differ significantly among treatments, indicating a limited translocation of Cd from roots to shoots.

As observed for Cd, root Hg concentrations exceeded those recorded in shoots across all treatments; however, the magnitude of this difference was markedly lower than that observed for Cd (Table 4). In shoots, Hg content increased significantly with rising concentration (*p* < 0.0001), reaching a maximum of 458.5 µg g^−1^ at 1.6 mg L^−1^. In contrast, roots exhibited a similar trend, although no significant difference was recorded at 1.2 and 1.6 mg L^−1^. BCF values in roots decreased from 536.4 ± 12.1 to 332.8 ± 5.0 with increasing Hg concentration, whereas shoot BCF remained stable across treatments (Table 4). Notably, TF values increased significantly with rising Hg supply, indicating that at higher concentrations a proportionally greater fraction of absorbed Hg was translocated to shoot tissues. Nevertheless, TF values remained below 1.0 across all treatments, as also observed for Cd.

### 2.2. Oxidative Stress Markers Under Cd and Hg Stress

Lipid peroxidation was evaluated by determining malondialdehyde (MDA) content in shoots of *A. subterranea*. Cd exposure induced a significant increase in MDA levels compared to the control (*p* < 0.0001). The maximum value recorded was 2.98 μmol MDA mg^−1^ protein for the 6 mg L^−1^ Cd treatment (Figure 1A). Hg exposure likewise resulted in a significant elevation in MDA content compared to control plants (*p* < 0.0001), reaching a maximum of 2.31 µmol mg^−1^ protein at 1.2 mg L^−1^ Hg (Figure 1B).

To further characterize the oxidative stress response of *A. subterranea*, H_2_O_2_ content was determined in shoots following 8 weeks of exposure to Cd and Hg. Under Cd treatment, a significant rise in H_2_O_2_ was observed compared to the control (*p* < 0.0001). The values showed increases ranging from 34.5% to 54.6% (Figure 2A). A similar trend was recorded under Hg exposure, where H_2_O_2_ content increased significantly across all treatments relative to the control (*p* = 0.0004), rising from 0.32 nmol mg^−1^ fresh weight (FW) in the control to approximately 0.97 nmol mg^−1^ FW at 1.6 mg L^−1^, a nearly three-fold increase (Figure 2B).

### 2.3. Antioxidant Enzyme Activity and Glutathione Metabolism Under Cd and Hg Stress

Catalase (CAT) activity in shoots of *A. subterranea* exhibited distinct response patterns depending on the metal evaluated. Under Cd exposure, a significant increase in CAT activity was observed at 3 mg L^−1^ (*p* = 0.0259), with values rising approximately 16.5% above the control. However, at higher Cd concentrations, CAT activity declined to levels comparable to the control (Figure 3A). In Hg treatments, CAT activity exhibited a fluctuating pattern, with significant increases of 20.6% and 37.6% at 0.8 mg L^−1^ and 1.6 mg L^−1^ Hg, respectively (*p* = 0.0002; Figure 3B).

Ascorbate peroxidase (APX) activity in shoots increased significantly under both Cd and Hg exposure. Under Cd exposure, APX activity increased significantly (*p* < 0.0001), showing enhancements ranging from 126% to 255% relative to the control (Figure 3C). A comparable response was observed under Hg exposure, where APX activity increased significantly across all treatments (*p* < 0.0001), reaching a maximum of 1.27 µmol asc mg^−1^ prot min^−1^ at 1.6 mg L^−1^ (Figure 3D).

Consistent with the upregulation of APX activity, glutathione (GSH) content also increased significantly under both metals. In response to Cd treatment, a significant accumulation of GSH was detected with increasing Cd supply (*p* < 0.0001), reaching a maximum of 0.1989 nmol GSH g^−1^ FW at 6 mg L^−1^ (Figure 4A). Under Hg exposure, the increase in GSH content was particularly pronounced, reaching 250–340% above control values at 1.2 and 1.6 mg L^−1^, respectively (*p* < 0.0001; Figure 4B).

## 3. Discussion

Cadmium and mercury toxicity severely compromises plant survival and growth performance, generally leading to significant reductions in biomass accumulation [24,25,26]. In the present study, *Adesmia subterranea* exposed to increasing Cd concentrations showed a marked reduction in shoot and root biomass, with responses inversely proportional to metal concentration. These results agree with previous studies demonstrating that Cd stress significantly impairs plant growth and biomass accumulation [27,28]. In contrast, Hg did not affect survival, and biomass showed no concentration-dependent inhibition. Shoot and root biomass decreased only at the lowest concentration (0.8 mg L^−1^) and did not differ from the control at 1.2 and 1.6 mg L^−1^, corroborating evidence from other species including *Trifolium repens* L. [29], *Brassica juncea* (L.) Czern. genotypes [30], *Artemisia lavandulaefolia* DC. [31], *Acacia mangium* Willd. [32] and *Jatropha curcas* L. [33] in which Hg tolerance was similarly maintained under comparable exposure conditions. This pattern is also consistent with our previous findings at the early seedling stage, where Hg exposure showed no inhibitory effect in *A. subterranea*, in contrast to the marked phytotoxicity of Cd [3].

The uptake and translocation of HMs in plants vary widely among metals and species, and they depend on factors such as plant genotype, soil physicochemical properties, and metal bioavailability [34,35]. In the present study, *A. subterranea* showed a strong capacity to accumulate both Cd and Hg in shoot and root tissues, reaching shoot Cd concentrations of 456.4 µg g^−1^ at 6 mg L^−1^, with shoot Hg concentrations attaining comparable levels (up to 458.5 µg g^−1^), showing a significant, dose-dependent accumulation pattern. Despite these elevated shoot concentrations, Cd was consistently retained at much higher levels in roots, confirming the primary role of the root system as the main site of Cd sequestration, a pattern widely documented for Cd in hybrid rice varieties [36], *Phytolacca acinosa* Roxb. [37], *Allium ramosum* L., *Imperata cylindrica* L., *Artemisia frigida* Willd., *Convolvulus arvensis* L. [38] and *Conyza canadensis* L. [39]. For Hg, concentrations were also higher in roots than in shoots, as reported in *Emilia fosbergii* Nicolson [40], *Fagopyrum tataricum* (L.) Gaerth. [41], *Brachiaria decumbens* Stapf [42] and *Medicago sativa* L. [43]. However, the root-to-shoot difference narrowed with increasing dose at 1.6 mg L^−1^; shoot and root concentrations reached 458.5 and 532.5 µg g^−1^, respectively, indicating that root retention of Hg was considerably weaker than that of Cd at the highest dose.

When we analyzed the TF, a difference between the two HMs was observed: the Cd TF stayed low and flat (<1) across the concentration range, whereas the Hg TF showed an overall increase with increasing Hg dose. The maximum value (0.79) is close to the average TF (0.72) reported for Hg-tolerant *Tridax procumbens* L. [44]. Notably, this pattern is not shared by its congener *A. pinifolia*, where the TF remained stable across all evaluated doses [21], suggesting that the saturation behavior seen here may be specific to *A. subterranea* rather than a general feature of the genus. The strong root retention of Cd may be attributed to rapid root uptake coupled with slower transport to aerial tissues [45,46]. Restricting metal transport to shoots, together with binding to cell wall components, chelation by thiol-rich ligands and vacuolar sequestration, is a recognized tolerance strategy in metal-tolerant plants [47] and a prerequisite for phytostabilization, where metals must be immobilized in roots while the plant maintains physiological function. The combination of BCF > 1 and TF < 1, observed for both HMs, indicates efficient uptake and, in the case of Cd, restricted translocation to shoots. For Hg, TF values approached 1 at the highest dose, so its retention in roots appears less stable. Taken together, these findings suggest that *A. subterranea* has potential for phytostabilization of Cd and low to moderate Hg-contaminated soils in semi-arid high-Andean environments as it combines metal retention in roots with tolerance mechanisms commonly associated with this strategy [47].

Cd and Hg toxicity disrupts key metabolic processes and pathways, leading to excessive production of ROS and consequent oxidative stress [48,49]. In the present study, *A. subterranea* exhibited a significant increase in H_2_O_2_ content under both metal treatments, reaching above control values for Hg and Cd, respectively, indicative of impaired cellular redox homeostasis. Although Cd is not a redox-active metal, it indirectly promotes ROS generation by interfering with electron transport chains and cellular antioxidant systems. Comparable H_2_O_2_ accumulation and oxidative stress responses under Cd and Hg exposure have been reported in a range of species, including *Oryza sativa* L., *Prosopis flexuosa* DC. f. flexuosa, *Larrea cuneifolia* Cav., *Chenopodium quinoa* Willd., and *Triticum aestivum* L. [25,50,51,52].

Consistent with enhanced ROS production, MDA content increased proportionally under both Cd and Hg exposure, reaching approximately 200–350% above control values at the highest treatment levels. As a well-established biomarker of lipid peroxidation, MDA reflects oxidative damage to membrane lipids, which are among the first cellular structures compromised under heavy metal stress [51].

The parallel elevation of H_2_O_2_ content and MDA accumulation supports the hypothesis that Cd and Hg not only stimulate ROS generation but also trigger lipid peroxidation, compromising membrane integrity in *A. subterranea*. Similar oxidative responses have been widely reported in *Erigeron canadensis* L. [53], in addition to Hg-related lipid peroxidation in *Fagopyrum tataricum* [41] and *Salvia limbata* C.A.Mey. [54].

Under HM stress, antioxidant enzyme activities generally increase with rising metal concentrations; however, excessive exposure can impair enzymatic defenses, ultimately leading to a decline in enzyme activity [55]. In *A. subterranea*, CAT activity increased by 16.5% at 3 mg L^−1^ Cd, indicating early activation of H_2_O_2_ detoxification mechanisms under moderate stress. At higher Cd concentrations, however, CAT activity declined to control levels, suggesting enzyme inhibition or structural damage induced by excessive ROS accumulation or displacement of essential HM cofactors by Cd ions [56]. Similar inhibitory effects of Cd on CAT have been reported in other species, where elevated Cd concentrations suppressed activity despite initial induction [27,51].

In contrast, APX activity increased markedly under Cd exposure, rising by 126% to 255% relative to the control as metal concentration increased. This compensatory upregulation of APX activity has been reported in seedlings of *Oryza sativa* cv. BRRI dhan29 [57] and *Trigonella foenum-graecum* L. [58], where APX assumed a dominant role in H_2_O_2_ detoxification when CAT efficiency declined. APX is generally considered more efficient than CAT in detoxifying H_2_O_2_ under metal stress, possibly owing to its several subcellular isoforms and its integration with the ascorbate–glutathione cycle [47]. Such functional compensation reflects the flexibility of the ascorbate–glutathione cycle in maintaining redox homeostasis under metal stress [59]. Together, these results indicate a shift in antioxidant response in *A. subterranea* with increasing Cd concentration. CAT contributes to H_2_O_2_ detoxification under moderate stress, while APX activity increased most markedly at higher Cd concentrations.

Unlike the pattern observed under Cd exposure, both CAT and APX activities were induced from the lowest Hg concentration tested (by more than 20% and 147% relative to the control, respectively). This activation of enzymatic defenses suggests a more marked antioxidant response to Hg stress than to Cd. This activation of enzymatic defenses suggests a stronger antioxidant response to Hg stress than to Cd. Comparable enhancements in CAT and APX activity under Hg exposure have been reported in *Lemna minor* L. [60], *Lepidium sativum* L. [61], *Cichorium intybus* L. [62], *Fagopyrum tataricum* (L.) Gaertn. [41] and *Solanum lycopersicum* L. [63], reinforcing the consistency of this response across taxonomically diverse species. In contrast, *A. pinifolia* exhibited inhibition of CAT and APX under Hg exposure [21], indicating species-specific variability in enzymatic antioxidant responses and a limited contribution of these defenses in that species.

In addition to enzymatic defenses, GSH, a central non-enzymatic antioxidant and metal chelator, was significantly elevated under both metal treatments, reaching levels 300–340% above control values at the highest Cd and Hg concentrations, respectively, a response also documented in *A. pinifolia* [21]. Comparable GSH accumulation has been reported under Cd stress in *Triticum aestivum* L. and *Youngia japonica* (L.) DC. [64,65] as well as in *Arundo donax* L., *Brassica juncea* and *Cyrtomium macrophyllum* (Makino) Tagawa under Hg exposure [66,67,68]. The elevated GSH levels likely contribute both to direct ROS scavenging and to the formation of Cd and Hg thiol complexes, facilitating intracellular metal sequestration and reducing metal-induced cellular damage [69]. The concurrent increase in APX activity and GSH accumulation is consistent with the involvement of both enzymatic H_2_O_2_ detoxification and non-enzymatic Cd/Hg chelation, although time-course data would be needed to establish the relationship between them. Together with the restricted translocation of Cd and Hg to shoots, this antioxidant response is consistent with the potential for phytostabilization of *A. subterranea* under metal exposure since efficient ROS scavenging and metal chelation allow plants to sustain growth while retaining metals in root tissues [47].

This study represents a first step toward identifying native species with potential for application in mining-impacted areas, addressing a substantial knowledge gap that currently exists for Argentina in this field. The experiments were conducted in a semi-hydroponic system under single metal exposure over a single time period, which does not fully capture the complexity of field soil or multi-metal conditions typical of mine tailings. Longer-term, soil-based trials with combined Cd–Hg exposure are needed to validate these findings under realistic field conditions.

## 4. Materials and Methods

### 4.1. Plant Material and Growing Conditions

*Adesmia subterranea* Clos, commonly called “Cuerno de Cabra,” is a high-Andean cushion shrub reaching up to 50 cm tall. It is found at elevations between 3200 and 4000 m a.s.l. and naturally distributed along the Central Andes Mountains of La Rioja, Mendoza, and San Juan (Argentina), as well as in Chile [70]. Seeds used in this study were collected from the Manzano Historico Nature Reserve (Mendoza, Argentina).

Dormancy was broken following Parera and Ruiz [71]. Seeds were soaked in concentrated sulfuric acid (H_2_SO_4_) for 5 min and rinsed with tap water. Then, they were sown in a perlite semi-hydroponic system in a growth chamber under controlled conditions. The growth conditions were 20 ± 2 °C with a 12-h light/12-h dark photoperiod (photosynthetic photon flux density PPFD 200 μmol/m^2^/s) and 50–55% relative humidity for 8 weeks.

To ensure optimal nutrient availability, the test solutions (including heavy metal treatments and nutrient solutions) were replaced every 3 days. The Hoagland nutrient solution included the following: 0.75 mM KNO_3_; 0.325 mM Mg(NO_3_)_2_·6H_2_O; 10 µM KH_2_PO_4_; 8 µM H_3_BO_3_; 0.2 µM MnSO_4_·H_2_O; 0.2 µM ZnSO_4_·7H_2_O; 0.2 µM CuSO_4_·5H_2_O; 0.2 µM (NH_4_)_6_Mo7O_24_·4H_2_O, 0.85 mM CaSO_4_·2H_2_O [21]. All experimental treatments, including the control, received Hoagland nutrient solution.

For each treatment, three independent replicates were established, each consisting of one 25-cell tray (50 cc per cell) sown with one seed per cell (25 seeds per tray). The tray was considered the experimental unit (*n* = 3 per treatment). For biomass, metal content and biochemical determinations, plants from the same tray were pooled to obtain one composite sample per tray (biomass: pool of six plants per tray), and samples were never mixed across trays. The six plants were selected at random.

### 4.2. Heavy Metal Treatments

The heavy metal treatments were carried out according to Parera et al. [21]. Stock solutions of Cd (as CdCl_2_·H_2_O) and Hg (as Hg(NO_3_)_2_·H_2_O) were used to prepare three treatment concentrations per metal: Cd at 3, 4.5, and 6 mg L^−1^ and Hg at 0.8, 1.2, and 1.6 mg L^−1^. A single control group (Hoagland nutrient solution without added metals) was shared by the Cd and Hg experiments, which were run simultaneously under the same conditions. The pH of all solutions was adjusted to 5.5 with 2 N HCl and measured at each renewal (every 3 days), being readjusted to 5.5 when necessary. Treatment concentrations were selected with reference to the soil quality guideline values for agricultural soils in Argentina (Law No. 24.051, “Régimen de Desechos Peligrosos”, Decree 831/93). These values were used to define the lowest exposure concentration for each HM, while the two higher concentrations represented 150% and 200% of the corresponding reference value.

### 4.3. Survival, Plant Growth and Accumulation Analysis of HMs

Survival and biomass were recorded after 8 weeks of treatment. The plants were harvested, separated into shoots (leaves and stems) and roots, cleaned and rinsed repeatedly in deionized water [72], and dried in an oven at 70 °C for 48 h. Biomass was recorded by pooling and weighing six plants per replicate.

Tissue Cd content was quantified by taking 0.1–0.3 g of plant material and oven-drying it until fully dry. The sample was then placed in a cold muffle furnace at 450 ± 20 °C. Concentrated HNO_3_ (1 mL) was added to the ash, which was allowed to dry and then returned to the muffle furnace. Finally, the ash was dissolved in 5–10 mL of 1N HCl. All samples were analyzed in triplicate. Cd concentrations were measured using an atomic absorption spectrometer with a graphite furnace (Shimadzu AA-7000-ASC-7000 autosampler, Tokyo, Japan) (detection limit of 0.05 μg L^−1^).

The total Hg content in the plant tissues was determined through wet digestion of oven-dried material at 60 °C to constant weight. Plant material (1 ± 0.05 g) was placed in a Teflon vessel, followed by the addition of 5 mL of redistilled water and 5 mL of concentrated HNO_3_ (65%). The wet digestion was carried out in a hot bath at 65 °C for 24 h. After digestion, the solution was transferred to a volumetric flask and diluted with redistilled water to a final volume of 25 mL. Hg concentration was then quantified using Cold Vapor Atomic Absorption Spectrometry (CVASS), with the Shimadzu AA-7000 (Tokyo, Japan).

The HM accumulation potential of *A. subterranea* was assessed by determining the shoot and root bioconcentration factor (BCF) and translocation factor (TF) [73].

BCF = [metal] shoots or roots/[metal] solution)

TF = [metal] shoots/[metal] roots

### 4.4. H_2_O_2_ and MDA Content

Fresh shoot samples for these assays were immersed in liquid nitrogen immediately after harvest and stored at −80 °C until analysis.

The H_2_O_2_ contents of shoots were determined according to Alexieva et al. [74]. The samples (100 mg) were homogenized in 5 mL of TCA (0.1%) under liquid nitrogen and centrifuged at 12.000 g for 15 min. Following centrifugation, 0.5 mL of the supernatant was mixed with 0.5 mL of 10 mM phosphate buffer (pH 7.0) and 1 mL of 1 M potassium iodide. Immediately, the absorbance was recorded at 390 nm using a UV–Vis spectrophotometer. The amount of H_2_O_2_ was determined using the extinction coefficient 0.28 µM^−1^ cm^−1^ and expressed as nmol mg^−1^ FW (fresh weight).

The MDA content was determined according to Heath and Packer [75]. Shoots were homogenized in 20% trichloroacetic acid at a ratio of 1:10 (*w*/*v*) and an equal volume of 0.5% thiobarbituric acid prepared in 20% trichloroacetic acid. The homogenate was incubated at 95 °C for 25 min, then cooled on ice for 2 min and centrifuged at 14,000 rpm for 10 min. The supernatant was collected and the amount of MDA (e 560 nm = 155 mM^–1^ cm^–1^) was estimated by measuring absorbance at 532 nm, with the absorbance at 600 nm subtracted to correct for unspecific turbidity. The MDA concentration was expressed as µmol/mg protein. Protein concentration was determined in the crude enzyme extract by Bradford using bovine serum albumin as the standard [76].

### 4.5. Enzymatic and Non-Enzymatic Activities

Among the enzymes known to be involved in ROS detoxification, CAT and APX were selected and their activities were determined in shoots. Antioxidant enzyme activities were assessed from extracts (100 mg) of shoots tissues. The extracts were obtained by grinding the frozen samples in liquid N2 using a pestle and mortar and buffer 0.1M Tris–HCl pH 8.0, 0.1M ethylenediaminetetra acetic acid (EDTA), 0.2% Triton X-100 (*v*/*v*), and 1 mM phenylmethanesulfonyl fluoride, in a ratio of 1:2 (w:v). The homogenates were centrifuged at 23.326 g for 30 min at 4 °C, and supernatants were used to determine different enzymatic activities.

The CAT activity (EC 1.11.1.6) was evaluated spectrophotometrically by measuring the rate of H_2_O_2_ disappearance at 240 nm [77]. The reaction mixture contained 50 mM potassium phosphate buffer (pH 7.0) and 10.5 mM H_2_O_2_. The reaction was run at 25 °C for 2 min after adding the enzyme extract. The rate of decrease in absorbance at 240 nm (E = 39.4 mM^−1^ cm^−1^) was used to calculate the enzyme activity. CAT activity was expressed in µmol H_2_O_2_ mg^−1^ protein min^−1^.

The APX activity (EC 1.11.1.11) was determined by assessing the rate of H_2_O_2_-dependent oxidation of ascorbic acid in a reaction mixture consisting of 50 mM phosphate buffer, pH 7.4, 5 mM ascorbic acid, and enzyme extract [78]. The reaction was initiated by adding 100 μL of 0.1 mM H_2_O_2_ and the oxidation rate of ascorbic acid was estimated by following the decrease in absorbance at 290 nm for 2 min. Protein concentration was determined spectrophotometrically using bovine serum albumin as a standard [78].

GSH was extracted from homogenized fresh shoots (100 mg) in 1 mL 0.1 N HCl (pH 2) and 1% (*w*/*v*) PVP through centrifuging for 20 min at 4000 g and 4 °C. The GSH content of supernatants was estimated by incubating a 100 μL aliquot with phosphate buffer (0.5 M H_2_PO_4_/K_2_HPO_4_, containing 0.1 g ascorbic acid, and 0.05 g glyoxylic acid, pH 6.8) for 5 min at 60 °C. The solution was cooled quickly on ice. Ellman reagent (DTNB solution; 0.1 mL) was added to all tubes, mixed, centrifuged at 12,000 rpm for 2 min, and absorbance was measured at 412 nm. A control tube containing only buffer and GSH standard was prepared from 4 mM GSH stock solution and diluted 1:10. Results were expressed as nmoles GSH g^−1^ FW [79].

### 4.6. Data and Statistical Analyses

Biomass, HM concentration and bioconcentration factor (BCF) were analyzed by two-way ANOVA with treatment (control, Cd and Hg dosis) and type of organ (root and shoot) as fixed factors, including their interaction. Survival and antioxidant parameters were analyzed by one-way ANOVA with treatment as the only factor. All analyses were performed in GraphPad Prism version 8.0.0, with Tukey’s post hoc test applied at the 0.05, 0.01, 0.001 and 0.0001 probability levels. ANOVA assumptions were verified using Shapiro–Wilk (normality) and Levene (homogeneity of variance) tests.

## 5. Conclusions

The present study shows that *Adesmia subterranea* exhibits differential physiological responses to Cd and Hg stress under controlled conditions. Cd exposure reduced plant survival and caused a dose-dependent reduction in biomass accumulation, whereas Hg treatments did not affect survival and caused only a limited, non-concentration-dependent reduction in biomass.

Both metals were efficiently taken up (BCF > 1), but their distribution differed. Cd was consistently retained in roots with a low, stable TF (<1), whereas Hg showed a dose-dependent increase in translocation, indicating weaker root retention. This accumulation pattern was accompanied by an activation of antioxidant defenses, including CAT, APX, and GSH, which may have contributed to mitigating oxidative damage under HM stress. Overall, these results suggest that A. subterranea is a promising candidate for phytostabilization of Cd-contaminated soils, with a more limited, dose-dependent potential for Hg. These traits are particularly relevant in high-Andean mining environments, where native perennial species with deep root systems could contribute to the long-term stabilization of Cd- and Hg-contaminated soils, by reducing metal mobility and bioavailability.

Therefore, the present findings provide a preliminary indication that *A. subterranea* may be a suitable candidate for phytoremediation. However, further research is required to validate its performance, specifically, examining its behavior in soil environments across higher concentrations and combined Cd–Hg exposure, as well as longer exposure periods.

## Figures and Tables

**Figure 1:**
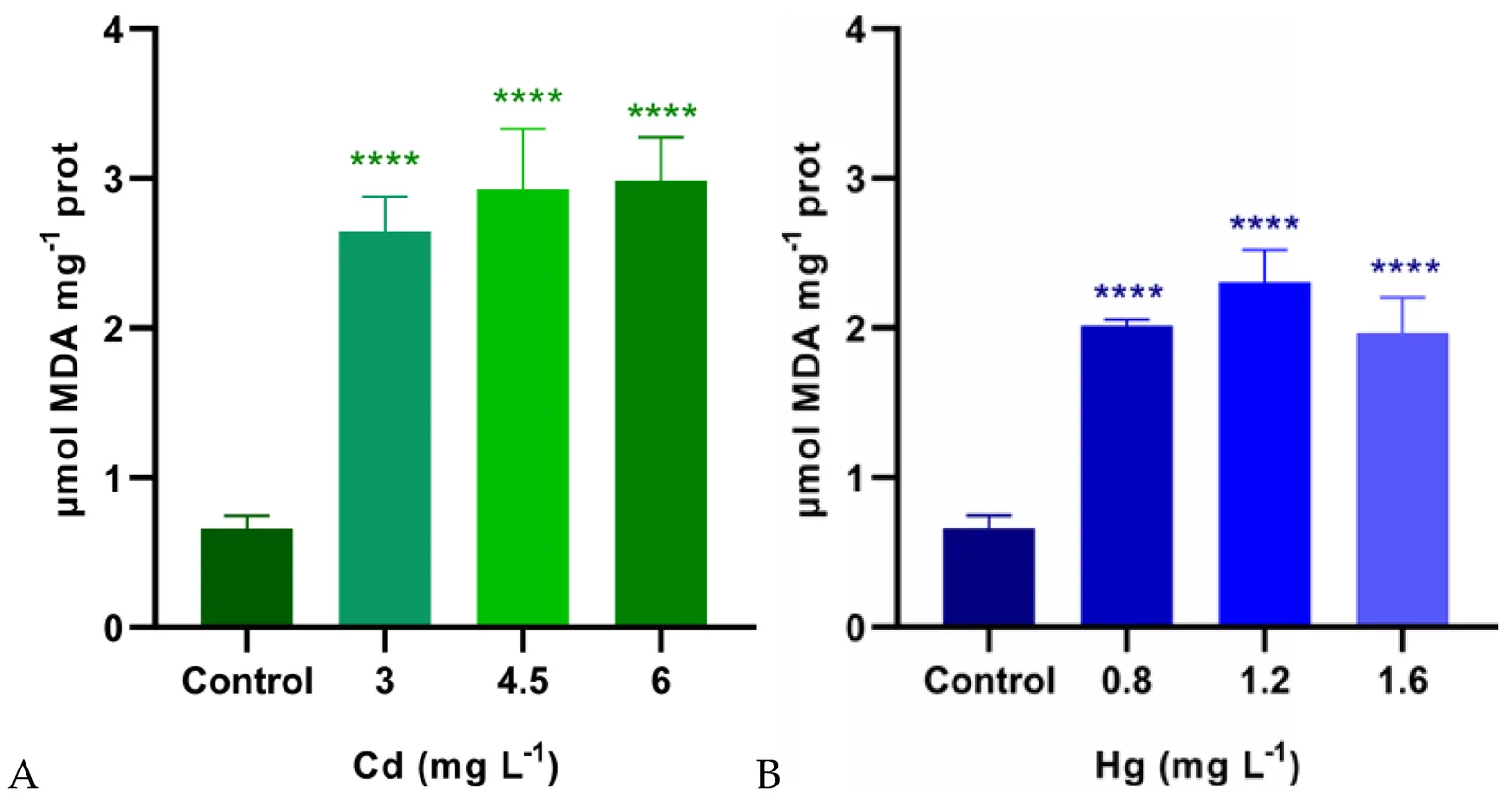
Shoot malondialdehyde (MDA) accumulation in *A. subterranea* after 8 weeks of (**A**) Cd (3, 4.5, and 6 mg L^−1^) or (**B**) Hg (0.8, 1.2, and 1.6 mg L^−1^) exposure. All values are means ± SD (*n* = 3). Significant differences at **** *p* < 0.0001 as compared to the control (Tukey’s post hoc test).

**Figure 2:**
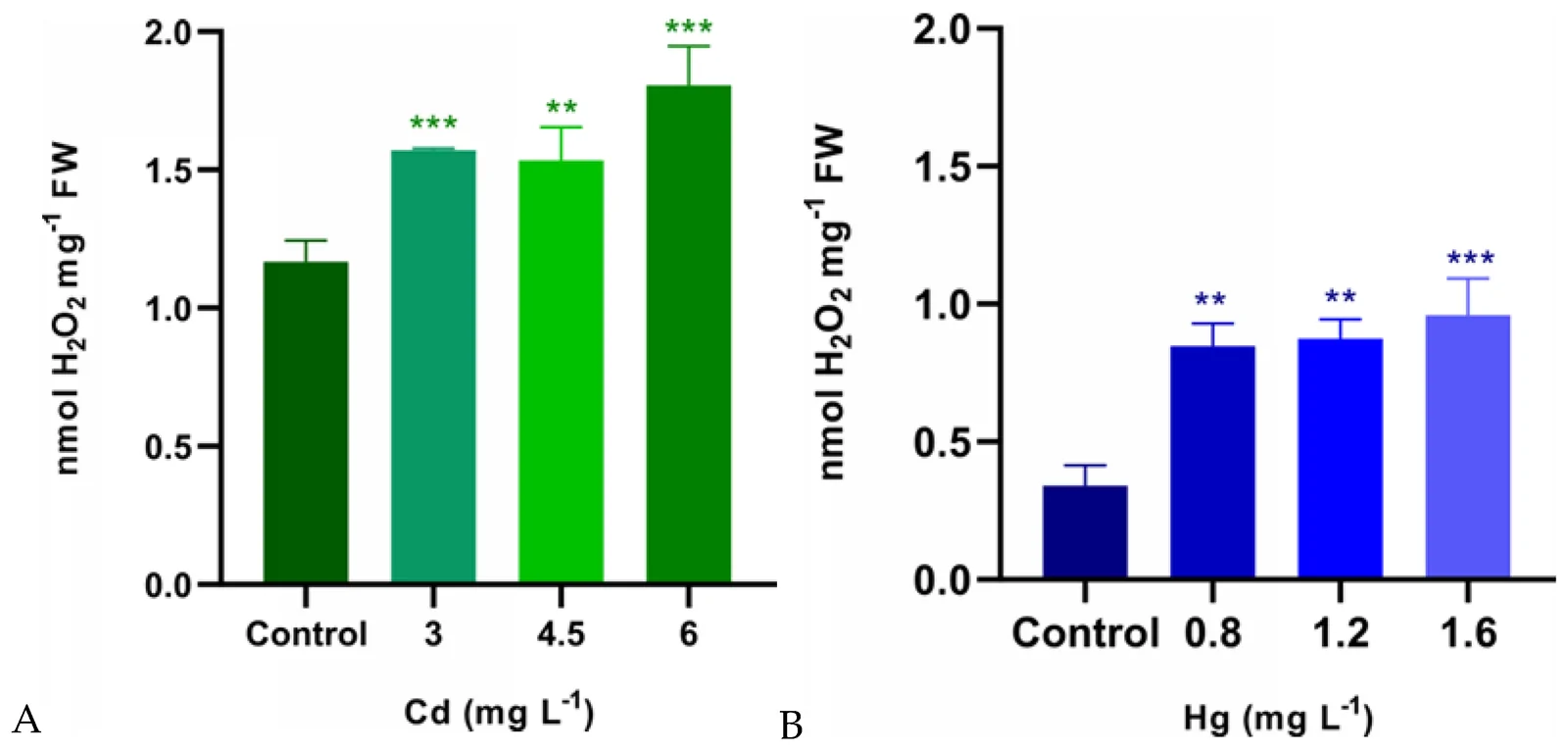
Shoot hydrogen peroxide (H_2_O_2_) accumulation in *A. subterranea* after 8 weeks of (**A**) Cd (3, 4.5, and 6 mg L^−1^) or (**B**) Hg (0.8, 1.2, and 1.6 mg L^−1^) exposure. Values represent means ± SD (*n* = 3). Significant differences at *** *p* < 0.001 and ** *p* < 0.01 as compared to the control (Tukey’s post hoc test); ns: not significant.

**Figure 3:**
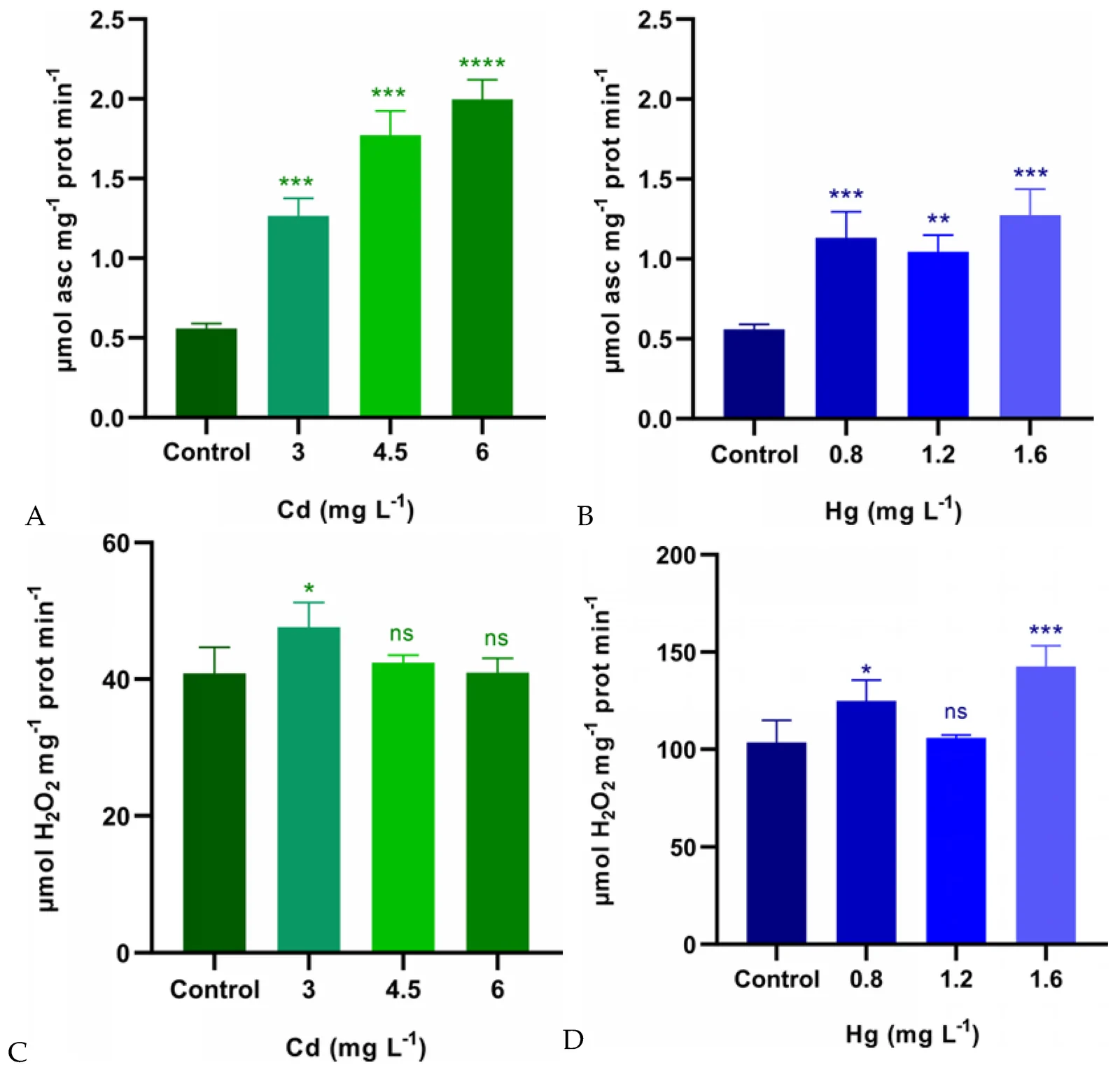
Catalase ((**A**,**B**) CAT) and ascorbate peroxidase ((**C**,**D**) APX) activities measured in *A. subterranea* shoots 8 weeks after Cd (3, 4.5, and 6 mg L^−1^) or Hg (0.8, 1.2, and 1.6 mg L^−1^) exposure. Data are means ± SD (*n* = 3). Significant differences at **** *p* < 0.0001, *** *p* < 0.001, ** *p* < 0.01, and * *p* < 0.05 as compared to the control (Tukey’s post hoc test); ns: not significant.

**Figure 4:**
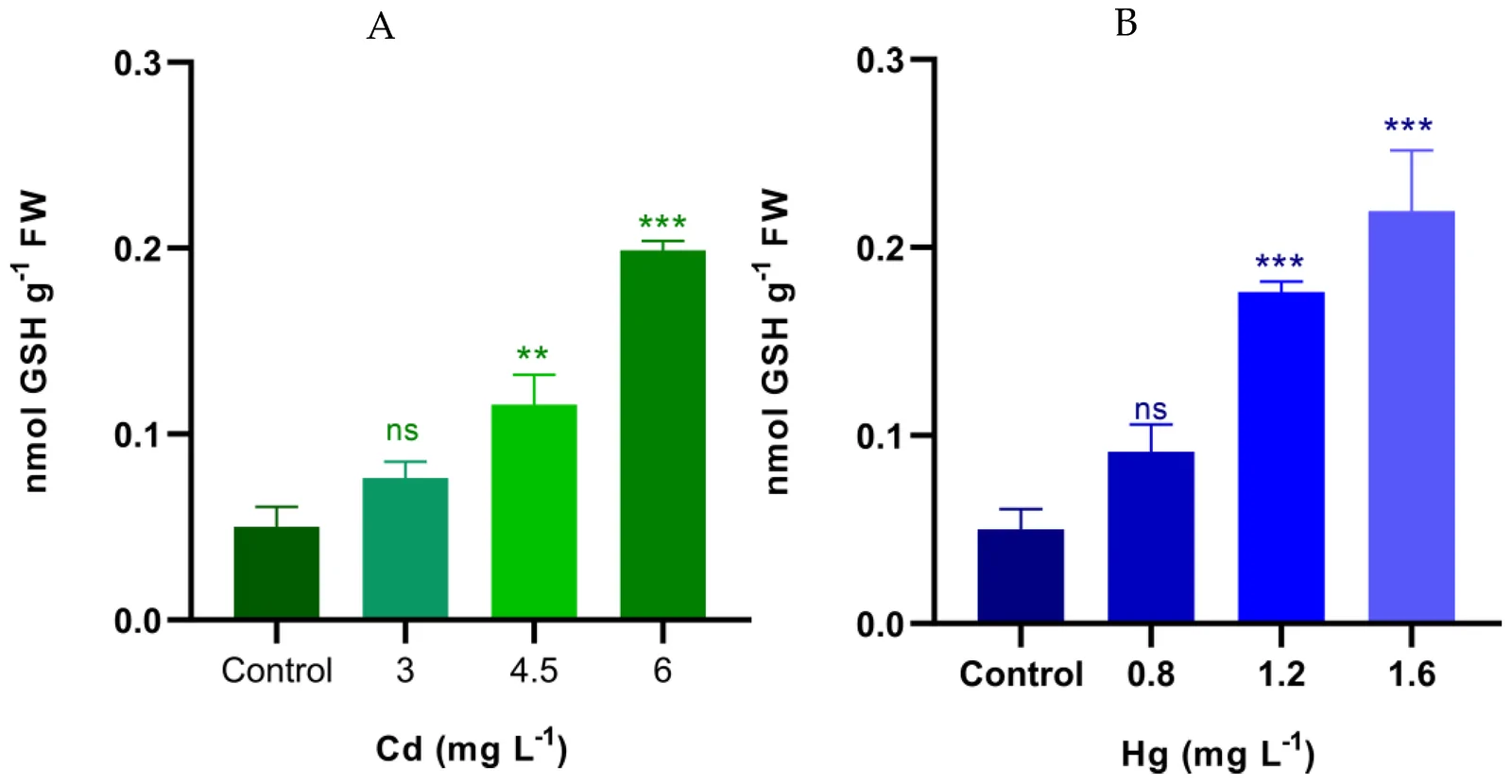
Glutathione (GSH) concentration in *A. subterranea* shoots 8 weeks after exposure to (**A**) Cd (3, 4.5, and 6 mg L^−1)^ or (**B**) Hg (0.8, 1.2, and 1.6 mg L^−1^). Data are means ± SD (*n* = 3). Significant differences at *** *p* < 0.001 and ** *p* < 0.01 as compared to the control (Tukey’s post hoc test); ns: not significant.

**Table 1:** Survival rates and dry biomass recorded for *A. subterranea* plants exposed to Cd concentrations (3, 4.5, and 6 mg L^−1^) and control (distilled water).

Treatment (mg L^−1^)		Survival (%)	Shoot Biomass (mg)	Root Biomass (mg)
Control		90 ± 5 ^a^	17.68 ± 1.41 ^a^	15.70 ± 1.55 ^a^
Cd	3	78.6 ± 2.3 ^b^	6.15 ± 0.98 ^b^	8.55 ± 2.00 ^b^
	4.5	74.6 ± 2.3 ^b^	3.94 ± 0.57 ^c^	5.61 ± 0.69 ^c^
	6	70.6 ± 8.0 ^b^	2.95 ± 0.29 ^d^	3.73 ± 0.70 ^d^

Note: Values shown are means ± SD of *n* = 3 (each replicate consisted of a pooled biomass of six plants). Different letters indicate significant differences between treatments (Tukey’s post hoc test *p* < 0.05).

**Table 2:** Survival rates and dry biomass recorded for *A. subterranea* plants exposed to Hg concentrations (0.8, 1.2 and 1.6 mg L^−1^) and control (distilled water).

Treatment (mg L^−1^)		Survival (%)	Shoot Biomass (mg)	Root Biomass (mg)
Control		90 ± 5 ^a^	17.68 ± 1.41 ^ab^	15.70 ± 1.55 ^a^
Hg	0.8	81.3 ± 6.1 ^a^	15.58 ± 2.39 ^c^	11.58 ± 1.23 ^b^
	1.2	83.3 ± 3.0 ^a^	18.54 ± 1.18 ^a^	14.84 ± 2.04 ^a^
	1.6	82.6 ± 3.2 ^a^	16.57 ± 1.36 ^bc^	15.0 ± 1.84 ^a^

Note: Values shown are means ± SD of *n* = 3 (each replicate consisted of a pooled biomass of six plants). Different letters indicate significant differences between treatments (Tukey’s post hoc test *p* < 0.05).

**Table 3:** Root and shoot Cd content (µg g^−1^), bioconcentration factor (BCF) and translocation factor (TF) in *A. subterranea* plants under Cd treatments (3, 4.5, and 6 mg L^−1^) and control.

Treatment (mg L^−1^)		Cd Concentration (µg g^−1^)		BCF		TF
		Shoots	Roots	Shoots	Roots	
Control		ND	ND	-	-	-
Cd	3	187.3 ± 15.7 ^a^	1016.3 ± 115.1 ^a^	62.4 ± 5.2 ^ab^	338.7 ± 38.3 ^a^	0.19 ± 0.03 ^a^
	4.5	242.2 ± 33.8 ^a^	1899.3 ± 142.3 ^b^	53.8 ± 7.5 ^b^	422.0 ± 31.0 ^ab^	0.19 ± 0.04 ^a^
	6	456.4 ± 50.5 ^b^	2885.4 ± 306.0 ^c^	76.0 ± 8.4 ^a^	480.9 ± 51.0 ^b^	0.16 ± 0.04 ^a^

Note: Different letters denote statistically significant differences between means (Tukey’s post hoc test, *p* < 0.05). ND: not detected.

**Table 4:** Root and shoot Hg content (µg g^−1^), bioconcentration factor (BCF), and translocation factor (TF) in *A. subterranea* plants under Hg treatments (0.8, 1.2, and 1.6 mg L^−1^) and control.

Treatment (mg L^−1^)		Hg Concentration (µg g^−1^)		BCF		TF
		Shoots	Roots	Shoots	Roots	
Control		ND	ND	-	-	-
Hg	0.8	223.6 ± 23.9 ^a^	429.1 ± 9.6 ^a^	279.6 ± 29.9 ^a^	536.4 ± 12.1 ^a^	0.48 ± 0.06 ^a^
	1.2	369.6 ± 28.5 ^b^	541.6 ± 22.3 ^b^	308 ± 23 ^a^	451.3 ± 18.5 ^b^	0.68 ± 0.05 ^b^
	1.6	458.5 ± 27.3 ^c^	532.5 ± 7.9 ^b^	286.6 ± 17.0 ^a^	332.8 ± 5.0 ^c^	0.79 ± 0.09 ^b^

Note: Different letters denote statistically significant differences between means (Tukey’s post hoc test, *p* < 0.05). ND: not detected.

## Data Availability

Data are included within the article. Further inquiries can be directed to the corresponding author.

## Abbreviations

The following abbreviations are used in this manuscript:

HM	Heavy metal
CAT	Catalase
APX	Ascorbate peroxidase
GSH	Glutathione
MDA	Malondialdehyde
BCF	Bioconcentration factor
TF	Translocation factor

## References

[1] Manwani S., Devi P., Singh T., Yadav C.S., Awasthi K.K., Bhoot N., Awasthi G. (2023). Heavy metals in vegetables: A review of status, human health concerns, and management options. Environ. Sci. Pollut. Res..

[2] Azizi M., Faz A., Zornoza R., Martinez-Martinez S., Acosta J.A. (2023). Phytoremediation potential of native plant species in mine soils polluted by metal (loid) s and rare earth elements. Plants.

[3] Parera V., Parera C.A., Feresin G.E. (2023). Germination and early seedling growth of high Andean native plants under heavy metal stress. Diversity.

[4] Rathod S.V., Saras P., Gondaliya S.M., Rathod S.V. (2024). Environmental pollution: Threats and challenges for management. Eco-Restoration of the Polluted Environment: A Biological Perspective.

[5] Oladoye P.O., Olowe O.M., Asemoloye M.D. (2022). Phytoremediation technology and food security impacts of heavy metal contaminated soils: A review of literature. Chemosphere.

[6] Rajendran S., Kamaludeen S.P.B., Subramanian A. (2024). Antioxidant defense mechanism in plants exposed to mercury toxicity: Response, tolerance and remediation. Earth and Environmental Sciences Library.

[7] Sarker A., Al Masud M.A., Deepo D.M., Das K., Nandi R., Ansary M.W.R., Islam A.R.M.T., Islam T. (2023). Biological and green remediation of heavy metal contaminated water and soils: A state-of-the-art review. Chemosphere.

[8] Latif N., Ahmed T. (2025). Different methods of industrial wastewater treatment: Industrial wastewater treatment. J. Chem. Mater. Sci. (JCMS).

[9] Gavrilescu M. (2022). Enhancing phytoremediation of soils polluted with heavy metals. Curr. Opin. Biotechnol..

[10] Priya A.K., Muruganandam M., Ali S.S., Kornaros M. (2023). Clean-up of heavy metals from contaminated soil by phytoremediation: A multidisciplinary and eco-friendly approach. Toxics.

[11] Diarra I., Kotra K.K., Prasad S. (2022). Application of phytoremediation for heavy metal contaminated sites in the South Pacific: Strategies, current challenges and future prospects. Appl. Spectrosc. Rev..

[12] Berrios L., Rentsch J.D. (2022). Linking reactive oxygen species (ROS) to abiotic and biotic feedbacks in plant microbiomes: The dose makes the poison. Int. J. Mol. Sci..

[13] Mohamed H.I., Ullah I., Toor M.D., Tanveer N.A., Din M.M.U., Basit A., Sultan Y., Muhammad M., Rehman M.U. (2025). Heavy metals toxicity in plants: Understanding mechanisms and developing coping strategies for remediation: A review. Bioresour. Bioprocess..

[14] Yaashikaa P.R., Kumar P.S., Jeevanantham S., Saravanan R. (2022). A review on bioremediation approach for heavy metal detoxification and accumulation in plants. Environ. Pollut..

[15] Jarin A.S., Khan M.A.R., Apon T.A., Islam M.A., Rahat A., Akter M., Anik T.R., Nguyen H.M., Nguyen T.T., Tran L.S.P. (2025). Plant responses to heavy metal stresses: Mechanisms, defense strategies, and nanoparticle-assisted remediation. Plants.

[16] Milla-Moreno E., Guy R.D. (2021). Growth response, uptake and mobilization of metals in native plant species on tailings at a Chilean copper mine. Int. J. Phytoremediation.

[17] de Castro Paes É., Veloso G.V., de Castro Filho M.N., Barroso S.H., Fernandes-Filho E.I., Fontes M.P.F., Soares E.M.B. (2023). Potential of plant species adapted to semi-arid conditions for phytoremediation of contaminated soils. J. Hazard. Mater..

[18] Plaza Cazón J., Benítez L., Murray J., Kirschbaum A., Kirschbaum P., Donati E. (2013). Environmental impact on soil, water and plants from the abandoned Pan de Azúcar Mine. Adv. Mater. Res..

[19] Lam E.J., Keith B.F., Montofré L., E Gálvez M. (2018). Copper Uptake by Adesmia atacamensis in a Mine Tailing in an Arid Environment. Air Soil Water Res..

[20] Lam E.J., Montofré Í.L., Ramírez Y. (2021). Mine tailings phytoremediation in arid and semiarid environments. Phytorestoration of Abandoned Mining and Oil Drilling Sites.

[21] Parera V., Pérez-Chaca M.V., Gallardo L.V., Gatica-Aguilar C., Parera C.A., Feresin G.E. (2024). Adesmia pinifolia, a native high-Andean species, as a potential candidate for phytoremediation of Cd and Hg. Plants.

[22] Roig F.A., Martinez Carretero E. (1998). La vegetación puneña en la provincia de Mendoza, Argentina. Phytocoenologia.

[23] Wang W., He Z., Dou D., Feng X., Liang X. (2026). Versatile roles of CrRLK1L proteins in crop adaptation to biotic and abiotic stresses. New Crops.

[24] Mei L., Zhu Y., Zhang X., Zhou X., Zhong Z., Li H., Li Y., Li X., Daud M.K., Zhu S. (2021). Mercury-induced phytotoxicity and responses in upland cotton (*Gossypium hirsutum* L.) seedlings. Plants.

[25] Dawood M.F., Sofy M.R., Mohamed H.I., Sofy A.R., Abdel-Kader H.A. (2023). N-or/and P-deprived *Coccomyxa chodatii* SAG 216–2 extracts instigated mercury tolerance of germinated wheat seedlings. Plant Soil.

[26] Song J., Sun Z., Saud S., Fahad S., Nawaz T. (2025). Exploring the deleterious effects of heavy metal cadmium on antioxidant defense and photosynthetic pathways in higher plants. Plant Stress.

[27] Hu Y., Wang H., Jia H., Peng M., Zhu T., Liu Y., Wei J. (2023). Effects of Cd treatment on morphology, chlorophyll content and antioxidant enzyme activity of *Elymus nutans* Griseb., a native plant in Qinghai-Tibet Plateau. Plant Signal. Behav..

[28] Aslam M.M., Okal E.J., Waseem M. (2023). Cadmium toxicity impacts plant growth and plant remediation strategies. Plant Growth Regul..

[29] Makarova A., Nikulina E., Tsirulnikova N., Avdeenkova T., Pishchaeva K.V. (2021). Potential of S-containing and P-containing complexones in improving phytoextraction of mercury by *Trifolium repens* L.. Saudi J. Biol. Sci..

[30] Ansari M.K.A., Ahmad A., Umar S., Zia M., Husen M.H., Owens A. (2021). Suitability of Indian mustard genotypes for phytoremediation of mercury-contaminated sites. S. Afr. J. Bot..

[31] Zhang G., Chen G., Yang R., Bai C., Yang W., Zhang J., Xuyang Y., Yang F., Seth C.S., Liu H. (2025). Concentration-dependent interactive toxicity of cadmium and mercury: Non-negligible effects on phytoremediation by indigenous *Artemisia lavandulaefolia*. Ecotoxicol. Environ. Saf..

[32] Sommer N., Guo Y., Rasche F., Hagemann M.H., Zörb C. (2026). The potential of four legume trees for mercury phytoremediation and the role of arbuscular mycorrhizal fungi. Environ. Sci. Pollut. Res..

[33] Domiciano D.R., Corrêa L.P., Matos L.R.H., Renken M., Consolin Filho N., de Araújo J.H.B. (2025). Phytoremediation of Mercury in Soil: Analysis of the Responses of *Mimosa pudica* L. and *Jatropha curcas* L.. Rev. Gestão Soc. Ambient..

[34] Shaari N.E.M., Tajudin M.T.F.M., Khandaker M.M., Majrashi A., Alenazi M.M., Abdullahi U.A., Mohd K.S. (2022). Cadmium toxicity symptoms and uptake mechanism in plants: A review. Braz. J. Biol..

[35] Zhao F.J., Tang Z., Song J.J., Huang X.Y., Wang P. (2022). Toxic metals and metalloids: Uptake, transport, detoxification, phytoremediation, and crop improvement for safer food. Mol. Plant..

[36] Luo Q., Bai B., Xie Y., Yao D., Zhang D., Chen Z., Zhuang W., Deng Q., Xiao Y., Wu J. (2022). Effects of Cd uptake, translocation and redistribution in different hybrid rice varieties on grain Cd concentration. Ecotoxicol. Environ. Saf..

[37] Dou C., Cui H., Zhang W., Yu W., Sheng X., Zheng X. (2023). Copper and cadmium accumulation and phytoextraction potential of native and cultivated plants growing around a copper smelter. Agronomy.

[38] Wang Q., Huang S., Jiang R., Zhuang Z., Liu Z., Wang Q., Wan Y., Li H. (2023). Phytoremediation strategies for heavy metal-contaminated soil by selecting native plants near mining areas in Inner Mongolia. Environ. Sci. Pollut. Res..

[39] Naz R., Khan M.S., Hafeez A., Fazil M., Khan M.N., Ali B., Javedd M.A., Imrana M., Shatie A.A., Alfaifie M.Y. (2022). Assessment of phytoremediation potential of native plant species naturally growing in a heavy metal-polluted industrial soils. Braz. J. Biol..

[40] Alves de Oliveira E., Cavalheiro da Silva L., Antônio de Andrade E., Dênis Battirola L., Lopes Tortorela de Andrade R. (2024). *Emilia fosbergii* Nicolson, a novel and effective accumulator for phytoremediation of mercury-contaminated soils. Int. J. Phytoremediation.

[41] Pirzadah T.B., Malik B., Tahir I., Irfan Q.M., Rehman R.U. (2018). Characterization of mercury-induced stress biomarkers in *Fagopyrum tataricum* plants. Int. J. Phytoremediation.

[42] Souza A.D.C., Jala I.M., Marques J.J., Januario J.P., Pires R.M.D.O., da Silva D.F., Souza Dias O.B., Vangronsveld J., Pozza A.A.A., Rabêlo F.H.S. (2025). *Brachiaria decumbens* for phytostabilization of mercury-polluted soils: From seed germination to plant growth. Int. J. Phytoremediation.

[43] Carrasco-Gil S., Ortega-Villasante C., Sobrino-Plata J., Barón-Sola Á., Millán R., Hernández L.E. (2023). Attenuation of mercury phytotoxicity with a high nutritional level of nitrate in alfalfa plants grown hydroponically. Plant Stress.

[44] de Oliveira E.A., Silva L.C.D., Battirola L.D., de Andrade R.L.T. (2025). *Tridax procumbens*: Applicable weed in phytoremediation and bioindication of soil contamination by mercury. ACS Omega.

[45] Alcantara H.J.P., Doronila A.I., Kolev S.D. (2017). Phytoextraction potential of *Manihot esculenta* Crantz. (cassava) grown in mercury-and gold-containing biosolids and mine tailings. Miner. Eng..

[46] Sterckeman T., Thomine S. (2020). Mechanisms of cadmium accumulation in plants. Crit. Rev. Plant Sci..

[47] Rai P.K., Sonne C., Kim K.H. (2023). Heavy metals and arsenic stress in food crops: Elucidating antioxidative defense mechanisms in hyperaccumulators for food security, agricultural sustainability, and human health. Sci. Total Environ..

[48] Anjum N.A., Duarte A.C., Pereira E., Ahmad I. (2014). Oxidative stress status, antioxidant metabolism and polypeptide patterns in *Juncus maritimus* shoots exhibiting differential mercury burdens in Ria de Aveiro coastal lagoon (Portugal). Environ. Sci. Pollut. Res..

[49] Noor I., Sohail H., Sun J., Nawaz M.A., Li G., Hasanuzzaman M., Liu J. (2022). Heavy metal and metalloid toxicity in horticultural plants: Tolerance mechanism and remediation strategies. Chemosphere.

[50] Bamagoos A.A., Alharby H.F., Abbas G. (2022). Differential uptake and translocation of cadmium and lead by quinoa: A multivariate comparison of physiological and oxidative stress responses. Toxics.

[51] Heredia B., Recio P.D., Young B.J., Pacheco P., Zungre F.B., Hruby S., Maria Andreína Acevedo M.A., Mercado M.I., Roqueiro G. (2025). Strategies of physiological, morpho-anatomical and biochemical adaptation in seedlings of native species exposed to mining waste. Ecotoxicol. Environ. Saf..

[52] Huang Y., Li F., Yi J., Yan H., He Z., Li X. (2022). Transcriptomic and physio-biochemical features in rice (*Oryza sativa* L.) in response to mercury stress. Chemosphere.

[53] Gan C., Liu Z., Pang B., Zuo D., Hou Y., Zhou L., Yu J., Chen L., Wang H., Gu L. (2022). Integrative physiological and transcriptome analyses provide insights into the Cadmium (Cd) tolerance of a Cd accumulator: *Erigeron canadensis*. BMC Genom..

[54] Kuru İ.S., Orcan P. (2025). Biochemical responses in *Salvia limbata* CA Mey. Towards mercury and lead induced in vitro oxidative stress. Sci. Rep..

[55] Chaâbene Z., Hakim I.R., Rorat A., Elleuch A., Mejdoub H., Vandenbulcke F. (2018). Copper toxicity and date palm (*Phoenix dactylifera*) seedling tolerance: Monitoring of related biomarkers. Environ. Toxicol..

[56] Luo P., Wu J., Li T.T., Shi P., Ma Q., Di D.W. (2024). An overview of the mechanisms through which plants regulate ROS homeostasis under cadmium stress. Antioxidants.

[57] Rahman A., Mostofa M.G., Nahar K., Hasanuzzaman M., Fujita M. (2016). Exogenous calcium alleviates cadmium-induced oxidative stress in rice (*Oryza sativa* L.) seedlings by regulating the antioxidant defense and glyoxalase systems: Calcium-induced cadmium stress tolerance in rice. Braz. J. Bot..

[58] Zayneb C., Bassem K., Zeineb K., Grubb C.D., Noureddine D., Hafedh M., Amine E. (2015). Physiological responses of fenugreek seedlings and plants treated with cadmium. Environ. Sci. Pollut. Res..

[59] Irshad M.A., Irfan A., Inam A., Zulfiqar U., Al-Hussain S.A., Sultan K., Afzal Hussain A., Al-Mutairi A.A., Rab Nawaz R., Zaki M.E. (2026). Synergistics effect of novel nano biochar on wheat growth, yield and antifungal response under cadmium contaminated soil: A field study. Ecotoxicol. Environ. Saf..

[60] Zhang T., Lu Q., Su C., Yang Y., Hu D., Xu Q. (2017). Mercury induced oxidative stress, DNA damage, and activation of antioxidative system and Hsp70 induction in duckweed (*Lemna minor*). Ecotoxicol. Environ. Saf..

[61] Smolińska B., Szczodrowska A. (2017). Antioxidative response of *Lepidium sativum* L. during assisted phytoremediation of Hg contaminated soil. New Biotechnol..

[62] Malik B., Pirzadah T.B., Tahir I., Ul Rehman R. (2019). Growth and physiological responses in chicory towards mercury induced in vitro oxidative stress. Plant Physiol. Rep..

[63] Bashir S., Jan N., Wani U.M., Raja V., John R. (2022). Co-over expression of Ascorbate Glutathione pathway enzymes improve mercury tolerance in tomato. Plant Physiol. Biochem..

[64] Qin S., Liu H., Nie Z., Gao W., Li C., Lin Y., Zhao P. (2018). AsA-GSH cycle and antioxidant enzymes play important roles in Cd tolerance of wheat. Bull. Environ. Contam. Toxicol..

[65] Yu B., Peng Y., Xu J., Qin D., Gao T., Zhu H., Zuo S., Song J., Dong J. (2021). Phytoremediation potential of *Youngia japonica* (L.) DC: A newly discovered cadmium hyperaccumulator. Environ. Sci. Pollut. Res..

[66] Xun Y., Feng L., Li Y., Dong H. (2017). Mercury accumulation plant *Cyrtomium macrophyllum* and its potential for phytoremediation of mercury polluted sites. Chemosphere.

[67] Raj D., Kumar A., Maiti S.K. (2020). *Brassica juncea* (L.) Czern. (Indian mustard): A putative plant species to facilitate the phytoremediation of mercury contaminated soils. Int. J. Phytoremediation.

[68] Li X., Zhao L., Teng Y., Luo Y., Zhao Q. (2023). Effects of cadmium on mercury accumulation and transformation by *Arundo donax* L. Environ. Sci. Pollut. Res..

[69] Shahid M., Pourrut B., Dumat C., Nadeem M., Aslam M., Pinelli E. (2014). Heavy-metal-induced reactive oxygen species: Phytotoxicity and physicochemical changes in plants. Rev. Environ. Contam. Toxicol..

[70] Kiesling R., de San Juan F. (1994). República Argentina: Vol.1, Pteridófitas Gimnospermas, Dicotiledóneas, Dialipétalas (Salicáceas a Leguminosas).

[71] Parera C.A., Ruiz M. (2003). Adesmia subterranea Clos germination physiology and pre-sowing treatments. J. Range Manag..

[72] Župunski M., Borišev M., Orlović S., Arsenov D., Nikolić N., Pilipović A., Pajević S. (2016). Hydroponic screening of black locust families for heavy metal tolerance and accumulation. Int. J. Phytoremediation.

[73] Zayed A., Gowthaman S., Terry N. (1998). Phytoaccumulation of trace elements by wetland plants: I. Duckweed. J. Environ. Qual..

[74] Alexieva V., Sergiev I., Mapelli S., Karanov E. (2001). The effect of drought and ultraviolet radiation on growth and stress markers in pea and wheat. Plant Cell Environ..

[75] Heath R.L., Packer L. (1968). Photoperoxidation in isolated chloroplasts: I. Kinetics and stoichiometry of fatty acid peroxidation. Arch. Biochem. Biophys..

[76] Bradford M.M. (1976). A rapid and sensitive method for the quantitation of microgram quantities of protein utilizing the principle of protein-dye binding. Anal. Biochem..

[77] Aebi H. (1984). Catalase in vitro. Methods in Enzymology.

[78] Chen G.X., Asada K. (1989). Ascorbate peroxidase in tea leaves: Occurrence of two isozymes and the differences in their enzymatic and molecular properties. Plant Cell Physiol..

[79] Akerboom T.P.M., Sies H. (1981). Assay of glutathione disulfide and glutathion mixed disulfides in biological samples. Methods Enzymol..

